# Comprehensive Insights into Metastasis-Associated Spinal Cord Compression: Pathophysiology, Diagnosis, Treatment, and Prognosis: A State-of-the-Art Systematic Review

**DOI:** 10.3390/jcm13123590

**Published:** 2024-06-19

**Authors:** Michail Vavourakis, Evangelos Sakellariou, Athanasios Galanis, Panagiotis Karampinas, Dimitrios Zachariou, Georgios Tsalimas, Vasileios Marougklianis, Evangelia Argyropoulou, Meletis Rozis, Angelos Kaspiris, Spiros G. Pneumatikos

**Affiliations:** 1 3rd Department of Orthopaedic Surgery, National & Kapodistrian University of Athens, KAT General Hospital, 14122 Athens, Greece; vagossak@hotmail.com (E.S.); athanasiosgalanis@yahoo.com (A.G.); karapana@yahoo.com (P.K.); dimitriszaxariou@yahoo.com (D.Z.); georgetsalimas@yahoo.com (G.T.); billmarou@gmail.com (V.M.); mrozhs@gmail.com (M.R.); angkaspiris@hotmail.com (A.K.); spirosgp@med.uoa.gr (S.G.P.); 2 Department of Orthopaedic Surgery, University General Hospital of Patra, 26504 Patras, Greece; eva.argirop@gmail.com

**Keywords:** spinal cord, compression, neurological impairment, cancer, metastases, review

## Abstract

**Background:** Spinal cord compression is a formidable complication of advanced cancer, and clinicians of copious specialities often have to encounter significant complex challenges in terms of diagnosis, management, and prognosis. Metastatic lesions from cancer are a common cause of spinal cord compression, affecting a substantial portion of oncology patients, and only in the US has the percentage risen to 10%. Acute metastasis-correlated spinal cord compression poses a considerable clinical challenge, necessitating timely diagnosis and intervention to prevent neurological deficits. Clinical presentation is often non-specific, emphasizing the importance of thorough evaluation and appropriate differential diagnosis. Diagnostic workup involves various imaging modalities and laboratory studies to confirm the diagnosis and assess the extent of compression. Treatment strategies focus on pain management and preserving spinal cord function without significantly increasing patient life expectancy, while multidisciplinary approaches are often required for optimal outcomes. Prognosis depends on several factors, highlighting the importance of early intervention. We provide an up-to-date overview of acute spinal cord compression in metastases, accentuating the importance of comprehensive management strategies. **Objectives:** This paper extensively explores the pathophysiology, clinical presentation, diagnostic strategies, treatment modalities, and prognosis associated with spinal cord metastases. **Materials and Methods:** A systematic literature review was conducted in accordance with the PRISMA guidelines. **Conclusions:** We aim to help healthcare professionals make informed clinical decisions when treating patients with spinal cord metastases by synthesizing current evidence and clinical insights.

## 1. Introduction

Acute spinal cord compression is a severe medical condition that can result from various aetiologies, including metastatic cancer, fractures, infections, vascular disorders, spondylosis, iatrogenic injuries, and developmental disorders [1]. Other common causes of spinal cord injury-associated compression are motor vehicle accidents, falls, interpersonal violence, and sports injuries [2]. Among all of these, metastatic lesions from cancer represent a significant portion of cases and are considered exceedingly exacting regarding management and treatment. Spinal cord metastases represent a critical manifestation of advanced cancer, significantly impacting patients’ quality of life and prognosis [1,2,3].

Spinal cord metastatic lesions affect 5% to 10% of the oncology patients in the USA [3]. Approximately 15% of all central nervous system lesions involve the spinal cord, with an incidence rate of 0.5–2.5 cases per 100,000 population [4]. Morbidity and mortality are influenced by the degree and level of spinal cord impairment [5]. The median survival for patients with spinal metastatic disease is around ten months [6]. People over fifty are more prone to back pain from metastatic tumours [7]. On the other hand, sarcomas and neuroblastomas are significant causes of metastatic spinal cord compression in children [7].

Metastatic cancer cells can spread to the spinal cord through arteries, more specifically retrograde through the Batson plexus, or by direct invasion through the intervertebral foramina [8], like in non-Hodgkin’s lymphoma where there is direct tumour extension. Deposition of tumour cells in the epidural space is common in leukaemia [7]. Common sites for spinal metastases are the thoracic spine (70%), lumbosacral spine (20%), and cervical spine (10%) [9]. In comparison, the primary sources of spinal metastases are the lung (31%), breast (24%), gastrointestinal tract (9%), prostate (8%), melanoma (4%), kidney (1%) [10], lymphoma (6%), and unknown origin (2%) [11]. Pain is ordinarily the initial symptom and can be either localized or radicular, but it is worsened by the Valsalva manoeuvre [12,13]. Compression by cancer masses also leads to weakness, sensory loss, and even paralysis [10,11,12,13].

Acute spinal cord compression, regardless of cause, can lead to oedema and diminished blood perfusion, potentially resulting in permanent neurological deficits if not promptly addressed [13,14]. Neurogenic shock, characterized by hypotension, bradycardia, peripheral vasodilation, and hypothermia, can occur above the T6 level due to sympathetic disruption. Spinal shock involves the complete loss of neurological function below the affected level, including autonomic dysfunction and reflexes. It is characterized by flaccid paralysis and can last hours to days, resolving when reflex arcs below the injury level resume function [14].

Examining the impact of particular studies on spinal metastases requires a thorough review of landmark research that has shaped the current understanding and treatment protocols. Patchell et al. (2005) found that direct decompressive surgery followed by radiotherapy significantly improved ambulatory outcomes and overall survival compared to radiotherapy alone. This established the importance of surgical intervention in select patients with spinal metastasis, particularly those with significant neurological deficits [15].

Palliative radiotherapy has been found to be effective in reducing pain and improving quality of life in patients with spinal metastasis. Thus, they reinforced the role of radiotherapy as a mainstay in the palliative management of spinal metastasis. Also, it has been demonstrated that bisphosphonates and denosumab reduce skeletal-related events and pain in patients with bone metastases, influencing guidelines on the use of bone-targeted therapies in managing spinal and other bone metastases [15].

The intricate nature of spinal cord involvement necessitates a comprehensive understanding of the underlying pathophysiology, optimal diagnostic approaches, and multidisciplinary treatment strategies. Prompt diagnosis and treatment are essential to preventing irreversible neurological deficits and improving patients’ quality of life. This article aspires to elucidate critical aspects of metastasis-associated spinal cord compression, providing clinicians with valuable insights to facilitate patient outcomes.

## 2. Materials and Methods

An extensive and rigorous systematic review was executed, scrutinizing all papers that were published until May 2024 investigating the metastasis-associated spinal cord compression syndrome. Publications from the following computerized databases were perused: MEDLINE/PubMed, Google Scholar, EMBASE, Web of Science, and Scopus. Keyword search terms were: “spinal cord”, “metastases”, “cancer”, “compression”, and “neurological impairment”. Language filters were activated for English. No restrictions were applied concerning the scientific articles’ publication dates. Article selection was performed independently by three authors, while disagreements were clarified with the assistance of two additional authors who made the final resolution. Inclusion criteria were clinical studies, case series, reviews, and papers reporting clinical cases regarding metastasis-associated spinal cord compression. On the other hand, exclusion criteria were articles not written in English, studies on spinal cord compression due to causes other than metastases, duplicate studies, unrelated case reports, previous review papers, and previous meta-analyses. Articles in their full text were scrutinized to retrieve additional relevant studies. The collected data were entered into an Excel 2021 spreadsheet. This systematic review is following the PRISMA guidelines, but was not registered.

## 3. Results and Discussion

**Figure 1:**
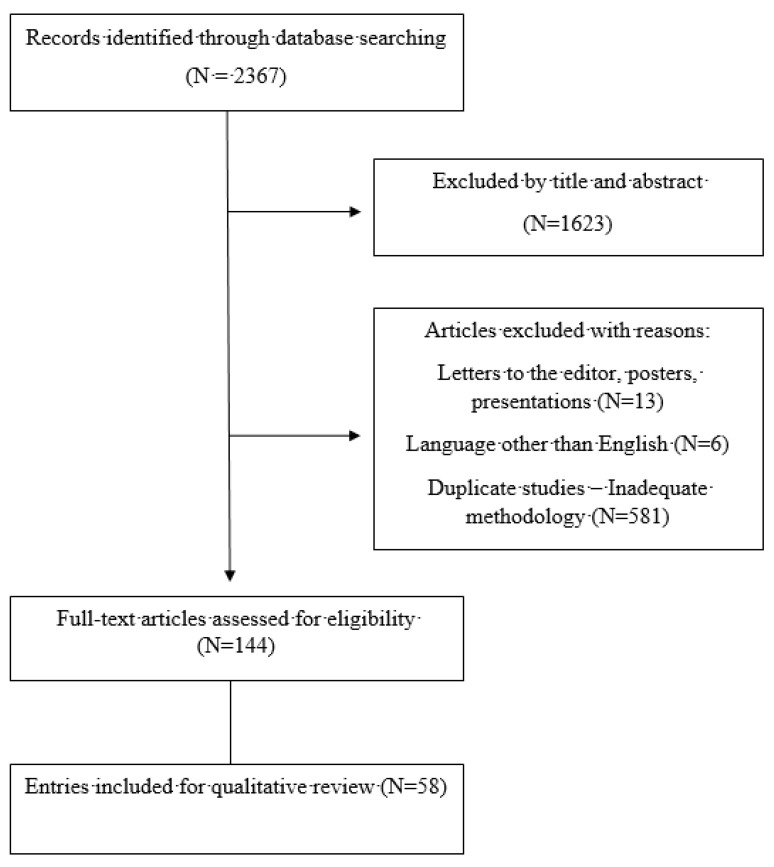
Study’s flowchart.

A total of 2367 papers were reviewed, and 1623 were excluded according to the title and abstract. Following that, 600 articles were excluded for other reasons. A total of 144 articles were selected for rigorous assessment. Finally, 58 papers were selected for citation (Figure 1).

### 3.1. Pathophysiology

The pathophysiology of spinal cord metastases involves multifaceted mechanisms, including hematogenous dissemination, direct invasion, and retrograde spread through Batson’s plexus [8]. Tumour cells infiltrate the vertebral column, leading to compression of neural structures, disruption of blood flow, and neurologic dysfunction. Additionally, inflammatory processes, altered blood perfusion, and oxidative stress contribute to the dynamic nature of spinal cord injuries, necessitating prompt intervention to mitigate progression [16].

### 3.2. Clinical Presentation

Early recognition of spinal cord metastases is paramount for timely intervention. The clinical presentation of acute spinal cord compression in metastases can be variable and non-specific (Table 1). Patients may present with atypical symptoms such as back pain, radicular pain, weakness, sensory deficits, and autonomic dysfunction. These symptoms may progress rapidly if left untreated, leading to severe neurological deficits and loss of function. A thorough history and physical examination are essential for diagnosing spinal cord compression.

Early symptoms include non-specific stiffness and/or pain, especially in patients with a known cancer history. Deteriorating back pain, particularly at night, and radicular pain suggest nerve root impingement from metastatic tumours [17]. Pain worsening in a recumbent position indicates vertebral metastasis [8,18]. Motor or sensory symptoms such as limb weakness or paraesthesia [19] and urinary or bowel dysfunction should raise suspicion of spinal cord compression [20]. Symptoms like difficulty climbing stairs, muscle stiffness, and urinary retention or constipation should prompt immediate attention for potential decompression [7].

### 3.3. Physical Examination

Physical examination plays a crucial role in identifying signs of spinal cord compression, including percussion tenderness, hyperreflexia, and motor or sensory deficits. However, it is essential to differentiate spinal cord compression from other conditions with similar presentations, such as brain neoplasms, infections, and mechanical back pain. Neurologic examination, including sensory testing and assessment of reflexes, aids in localizing spinal cord involvement and determining the extent of neurologic compromise. Furthermore, understanding the characteristic patterns of symptomatology associated with different tumour types facilitates accurate, pertinent diagnosis and treatment planning.

Physical examination findings include [21]: tenderness upon percussion in the affected spinal region, hyperreflexia, spasticity, and sensory loss, pain radiating down less symptomatic limb with straight leg raise, and absent or hypoactive deep tendon reflexes. The Babinski sign might be absent initially, and radicular pain is usually exacerbated by the Valsalva manoeuvre. Nuchal rigidity is present in 10% of patients with leptomeningeal metastases, while contralateral sensory and motor deficits may be found with lateral spinal cord compression. Additionally, cervical intramedullary tumours may present with isolated sensory loss in the upper extremities. Unfortunately, about half of tumour patients have paresis, and 15% are paraplegic at diagnosis, while foramen magnum metastases may cause quadriparesis initially.

Each region should be addressed separately: cervical region—test sensation in the upper extremities, focusing on dermatomes C4–T1; thoracic region—assess sensory function along the trunk, abdomen, and lower chest, covering dermatomes T1–T12; lumbar region—evaluate sensation in the lower abdomen, groin, and anterior thigh, examining dermatomes L1–L5; sacral region—test sensation in the buttocks, posterior thigh, and perineum, focusing on dermatomes S1–S5.

During a physical examination, clinicians identify the neurologic level of spinal cord damage by testing key muscles [16,22,23]: C5—elbow flexors, C6—wrist extensors, C7—elbow extensors, C8—long finger flexors, T1—small finger abductors, L2—hip flexors, L3—knee extensors, L4—ankle dorsiflexors, L5—long toe extensors, S1—ankle plantar flexors.

The ASIA impairment scale categorizes spinal cord damage [24]:

A. Complete loss of motor/sensory function in S4–S5;
B. Sensory, but not motor, function below affected level, including S4–S5;
C. Some motor functions below the affected level, most key muscles below that level grade <3;
D. Some motor functions below the affected level; most key muscles grade ≥3;
E. Normal motor and sensory function.

Complete injury: No motor/sensory function in lowest sacral segments. Incomplete injury: Some motor/sensory function preservation below injury level, including lowest sacral segments [25,26].

During physical exams to identify potential spinal cord compression from all routes, clinicians must assess pulmonary function by evaluating chest wall expansion, respiratory rate, and cough. In addition, assessing arterial blood gases and pulse oximetry for signs of hypoxia or carbon dioxide retention is vital. Respiratory dysfunction severity correlates with spinal cord injury level [27,28].

### 3.4. Diagnostic Evaluation

Diagnostic evaluation of spinal cord metastases relies on laboratory studies and imaging modalities. Elevated inflammatory markers, such as erythrocyte sedimentation rate, may provide clues to underlying malignancy. Imaging techniques, including MRI, CT, and myelography, offer detailed visualization of spinal cord lesions, facilitating precise localization and characterization of metastatic deposits. Neurological assessment, utilizing tools such as the ASIA impairment scale, further assists diagnostic and prognostic considerations [28,29,30,31].

Laboratory tests, such as erythrocyte sedimentation rate, complete blood cell count, and chemistry profile, can offer clues, but must be more definitive for a spinal cord neoplasm diagnosis. An elevated sedimentation rate suggests inflammation or infection, while a chemistry profile may hint at primary cancer [29].

All patients should undergo plain radiography for imaging studies, which is useful for detecting bony destruction (osteolytic or osteoblastic) [30], vertebral subluxation, collapse, and calcification. Owl-eye erosion in the lumbar spine AP view is characteristic of metastatic disease (90% of symptomatic patients). Moreover, osteoblastic changes are common in prostate cancer metastasis, Hodgkin’s disease, lymphoma, and breast cancer metastasis, and are detected in about 80% of spinal cord tumour patients [31].

**Figure 2:**
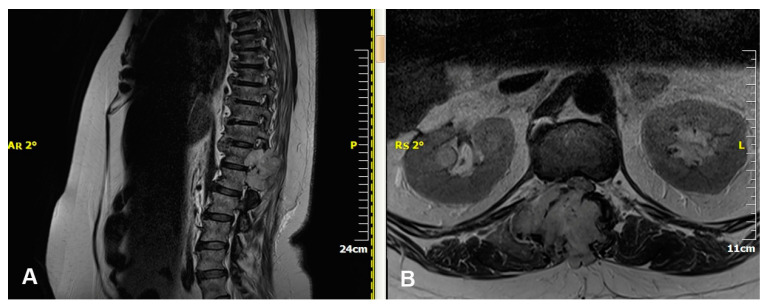
MRI of thoracolumbar renal metastasis. (**A**) Sagittal plane, (**B**) Axial plane.

**Figure 3:**
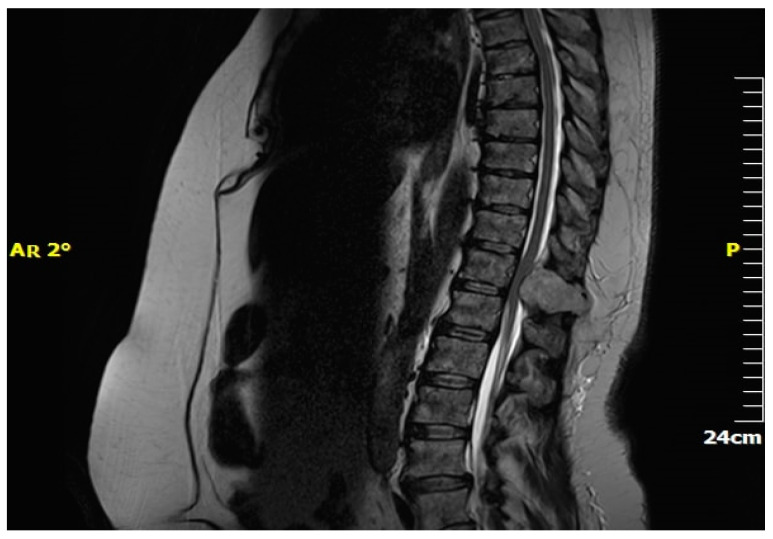
MRI—Spinal cord compression due to metastatic renal tumour.

Magnetic resonance imaging (MRI) is pivotal in the medical diagnosis of metastatic spine cancer [32] (Figure 2). MRI accurately detects spinal cord compression caused by masses, aiding in lesion definition and visualization of the entire spine (Figure 3). Contrast-enhanced images help to differentiate metastases from degenerative bone diseases. Diffusion-weighted images distinguish metastases from osteoporotic bones. MRI also aids in differentiating collapsed vertebrae due to trauma, osteoporosis, or malignant disease [33]. CT scanning complements MRI by delineating bony abnormalities and fractures, especially when plain radiography is inadequate [34,35]. Though it was once common, myelography has now been largely replaced by MRI and CT due to technological advancements [36].

Performing a biopsy for metastatic tumours in the spine is a critical step to establish a definitive diagnosis, identify the primary source of the tumour, and plan the appropriate treatment [37,38]. The timing and indications for a biopsy can vary depending on several factors. First of all, if imaging studies (e.g., MRI, CT, PET scans) reveal a spinal lesion and there is no known primary cancer, a biopsy is essential to determine the origin of the metastatic disease. Furthermore, when the imaging characteristics of the spinal lesion are not typical for a metastatic tumour, a biopsy is necessary to rule out other conditions such as primary spinal tumours, infections, or inflammatory processes. Also, if a patient with a known primary cancer develops new or worsening symptoms (e.g., severe pain, neurological deficits) and imaging suggests spinal metastasis, a biopsy may be performed to confirm the diagnosis and guide further treatment. Moreover, in cases where specific genetic or molecular markers are needed to tailor targeted therapies (e.g., targeted biologic agents or immunotherapy), obtaining tissue from the spinal lesion for biopsy can be crucial. Finally, for patients presenting with symptoms of spinal cord compression or vertebral instability without a known history of cancer, a biopsy is performed to diagnose the underlying malignancy [37,38].

### 3.5. Differential Diagnoses

Multiple pathologies can mimic spinal cord compression, like brain tumours, amyotrophic lateral sclerosis, spinal infections, Brown–Sequard syndrome, epidural hematoma, Cauda equina and conus medullaris syndromes, intervertebral disk issues, subdural and epidural infections, mechanical back pain, and vertebral fractures [39,40].

## 4. Treatment and Management

**Figure 4:**
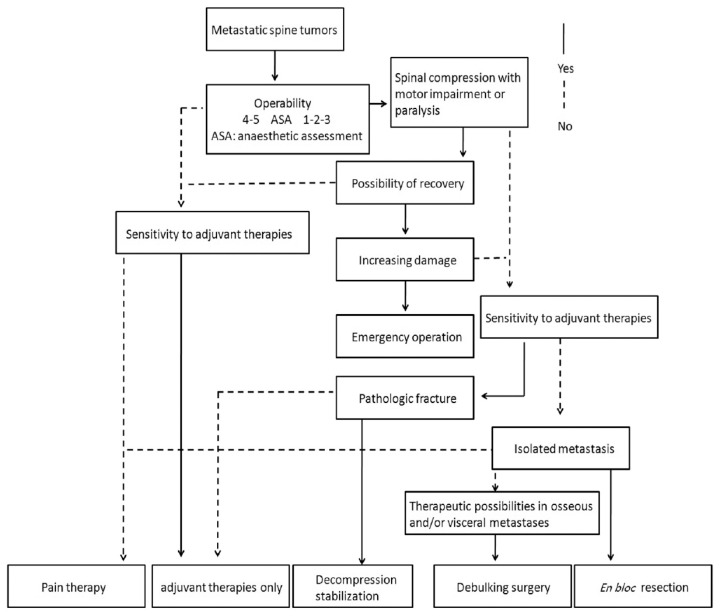
Algorithm for management of metastatic tumours.

The management of spinal cord metastases necessitates a multimodal approach tailored to individual patient characteristics and tumour burden. Treatment strategies (Figure 4) for acute spinal cord compression in metastases aim to relieve pain, preserve spinal cord function, and improve quality of life, even though they do not alter the patient’s life expectancy [41]. In the realm of treatment, both non-operative and operative approaches play crucial roles. Medical interventions, including corticosteroids, analgesics, and anti-neuropathic agents, aim to alleviate symptoms and optimize comfort. Surgical decompression, laminectomy, spinal stabilization, and minimally invasive procedures may be necessary in cases of severe compression or instability and, with tumour resection, may be indicated to relieve cord compression and preserve neurological function [42]. Additionally, radiation therapy plays a crucial role in tumour control and pain management, offering localized treatment while minimizing systemic toxicity [43]. Treatment choice depends on various factors, including the patient’s overall health status, the extent of tumour spread, and response to previous treatments [42,43]. Tailoring the treatment strategies to each patient’s unique circumstances is essential, prioritizing the no-harm principle. Combining therapeutic modalities, mainly radiotherapy and surgery, often yields the most favourable outcomes by synergistically addressing local disease control.

Moreover, there are new avenues for future research on the treatment of spinal metastasis. Immunotherapy investigates the efficacy and safety of immune checkpoint inhibitors (e.g., pembrolizumab, nivolumab) in treating spinal metastasis from various primary tumours using genetic profiling of spinal metastases to tailor targeted therapies based on the molecular characteristics of the tumour. Also, minimally invasive surgical techniques evaluate the long-term outcomes and quality of life in patients vs. open surgical techniques for spinal metastasis, which may reduce recovery time, complications, and healthcare costs while maintaining or improving outcomes. Furthermore, the synergistic effects of combining radiotherapy, chemotherapy, and novel agents like bisphosphonates or RANKL inhibitors in managing spinal metastasis may enhance tumour control and alleviate symptoms more effectively than single-modality treatment. Finally, artificial intelligence and machine learning can predict outcomes, personalize treatment plans, identify patients at high risk of complications, and analyse large datasets to uncover patterns and inform clinical decision making [37,38,44,45].

Bone pain in spinal cord compression can be managed with corticosteroids, primarily dexamethasone, which reduces oedema and cord compression [46]. Tapering doses gradually is essential to mitigate side effects like immunosuppression, gastrointestinal issues, and hyperglycaemia [47]. Monitoring blood glucose levels is crucial, especially for diabetic patients.

Except for bone pain, neuropathic pain is equally important. Antiepileptic drugs and tricyclic antidepressants are effective for neuropathic pain [48,49]. Topical preparations like lidocaine patches and opioids are additional options. Chemical epidural neurolysis is reserved for medically intractable pain due to its risks, especially in cases of structural instability and compression [50].

Another adverse effect of metastatic bone tumour is hypercalcemia, which can be managed by rehydration to address polyuria and pre-renal failure [51]. Steroids and bisphosphonates can be used to control the lytic process by inhibiting osteoclastic activity and reducing bone resorption [51].

### 4.1. Surgical Management

Tokuhashi et al. (2014) evaluated six prognostic systems, namely, the Bauer score, Katagiri score, Linden score, Rades score, Tokuhashi score, and Tomita score. These scoring systems each employ a unique set of factors to assess prognosis [44]. While the primary site of cancer and the presence of visceral metastasis are common factors among these systems, other variables differ between them. In their review, Tokuhashi et al. (2014) highlighted the significance and limitations of each scoring system, noting that they can only approximate the survival period. They also pointed out that despite these scores being used to guide surgical decisions and avoid unnecessary treatments, further research is needed. This includes incorporating more oncological perspectives and refining treatment processes. Aoude et al. noted that the revised Tokuhashi score could potentially be used to better estimate actual patient survival with certain modifications [45].

**Figure 5:**
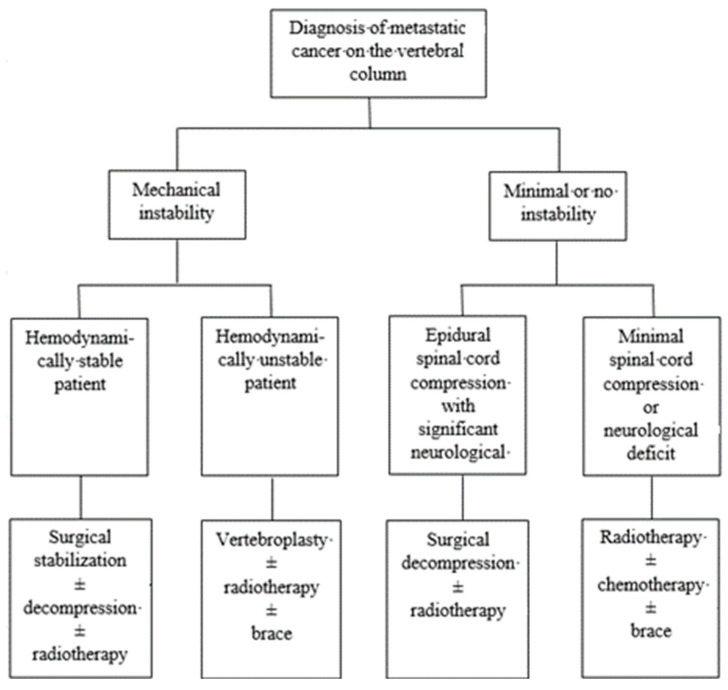
Algorithm for operative management of spinal metastases.

**Figure 6:**
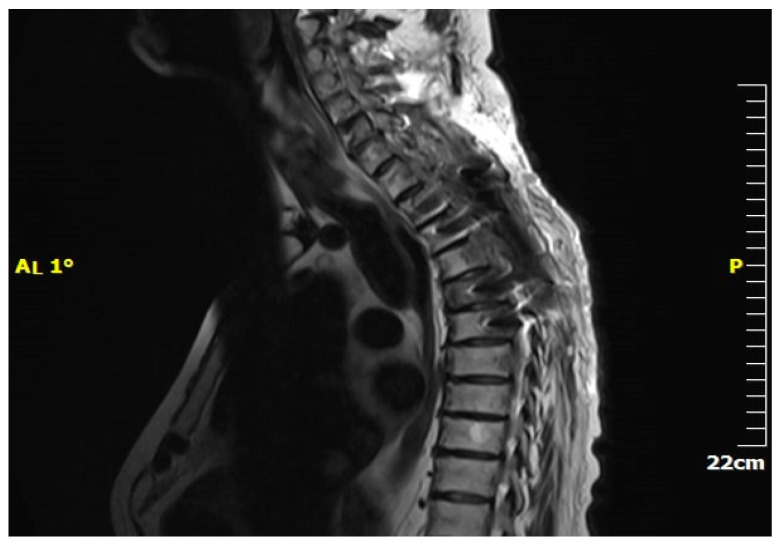
Post-surgical MRI—thoracic spinal fusion following mechanical instability caused by prostatic tumour metastasis.

The operative approach for spinal metastases involves stabilizing the affected vertebrae and relieving spinal cord compression (Figure 5). Radical surgical approaches aim to remove tumour masses while stabilizing the spine, often using techniques like spinal fusion with or without spondylectomy [52] (Figure 6). Other methods include laminectomy, minimally invasive endoscopic procedures, and kyphoplasty [53,54]. These techniques offer improved pain relief and functional recovery, especially when combined with stabilization procedures like pedicle screws.

Common surgical approaches include:

1. Transpedicular approach: This approach is used for tumours involving the dorsal aspect of the vertebral body, extending into the pedicle and associated dorsal elements [55].
2. Posterior approach: Allows early identification of the spinal cord and utilization of rigid or long constructs in posterior vertebral areas [56].
3. Costotransversectomy and lateral extracavitary approach: Posterior-lateral approaches provide access to the dorsal part of the affected vertebral body [8].

### 4.2. Radiation Therapy

Radiotherapy is the primary treatment for spinal cord compression due to metastases in radio-sensitive tumours, providing pain relief by reducing tumour size [57]. It is more effective (67%) than surgery (36%) for pain control, with surgery alone being the least effective, resulting in further deterioration for 20–26% of patients [58]. Treatment involves a multimodal approach, including steroids, radiation, and surgery, with minimally invasive surgery gaining popularity. Radiotherapy, typically 30–40 Gy over 2–4 weeks, targets the spinal cord based on its tolerance dose and tumour radiosensitivity [42]. Intensity-modulated radiation therapy (IMRT) and robotic linear accelerators improve precision and minimize damage to surrounding tissues. IMRT, in particular, allows for optimized, non-uniform radiation delivery, sparing healthy tissue [59]. Robotic LINACs offer further benefits with individual beam targeting, real-time tracking, and reference to anatomical landmarks [60].

### 4.3. Chemotherapy

Chemotherapy is indicated for tumours that are chemosensitive, such as Hodgkin’s disease and lymphoma. It can also be used alongside other treatments for prostate cancer, breast cancer, or multiple myeloma. The choice of chemotherapy agents depends on the tumour type and the patient’s history [61].

### 4.4. Complications

Despite advances in treatment, spinal cord metastases are associated with significant morbidity and mortality. Complications such as respiratory compromise, neurologic deficits, and treatment-related adverse effects may impact prognosis and quality of life [3]. Prognostic factors, including tumour histology, the extent of spinal cord involvement, and response to therapy, inform clinical decision making and prognostication. Fatal outcomes can result from spinal cord dysfunction and associated complications like renal failure, pulmonary embolism, pneumonia, or septicaemia. Prompt treatment is crucial to avoid severe consequences.

While autonomic dysfunction can severely impact an individual’s quality of life and self-efficacy, recovery is possible through comprehensive and individualized patient training programs. By focusing on education, physical rehabilitation, psychological support, and lifestyle modifications, patients can regain a sense of control over their lives, improve their symptoms, and enhance their overall well-being [62].

### 4.5. Prognosis

Timely diagnosis and treatment are crucial for patients with acute spinal cord compression due to metastases. Delay can lead to irreversible neurological deficits and a less than 5% chance of recovery if spinal cord injury occurs [63]. Despite advancements in treatment modalities, no definitive treatment has been shown to significantly increase life expectancy in patients with spinal metastases. However, aggressive management strategies aimed at pain control and preservation of neurological function can improve quality of life and benefit patients and their families [63]. Patients with uncontrolled pain from metastatic spinal cord compression often experience lower life satisfaction. Comprehensive supportive care, including symptom management and psychosocial support, is essential to optimize patient outcomes and enhance quality of life. The prognosis for patients with metastatic tumours and neurological deficits depends on the severity and duration of impairment at the start of treatment. Sphincter dysfunction indicates a poor prognosis [63]. The goal of therapy is symptom relief and maintaining independence. Educating patients about symptoms and the possibility of spinal cord compression is vital, especially for those with prostate, breast, or lung cancer [64]. Prompt reporting of changes in back pain is crucial to prevent neurological deficits.

## 5. Conclusions

Spinal metastasis, a common occurrence among cancer patients, presents a formidable challenge due to its potential to compress the spinal cord, leading to debilitating neurological symptoms. Diagnostic accuracy hinges upon comprehensive history-taking, meticulous physical examination, and judicious use of laboratory and imaging studies. These measures are indispensable in differentiating metastatic compression from other conditions and assessing the patient’s overall health status. Recognizing the urgency of timely intervention is paramount, as delayed treatment exacerbates neurological deficits and complicates the prospects of functional recovery. Although treatment options are available to alleviate symptoms and preserve spinal cord function, the prognosis remains variable and dependent on various factors. Overall, spinal cord metastases represent a complex clinical entity requiring a multidisciplinary approach to diagnosis and management. This article aims to empower clinicians with the knowledge and necessary tools to provide optimal care for patients with spinal cord metastases by elucidating critical aspects of pathophysiology, clinical presentation, diagnostic evaluation, treatment modalities, and prognosis. Future research on spinal metastasis should focus on innovative treatments, personalized medicine, and the integration of advanced technologies to improve patient outcomes. Continued exploration of the biological mechanisms underlying metastasis and treatment response will also be crucial in developing more effective therapies.

## 6. Overall Message

Understanding and treating spinal metastasis is crucial for alleviating human suffering. Timely intervention is essential to prevent severe neurological complications. The ultimate goal is to improve patients’ quality of life through comprehensive care and education.

## Figures and Tables

**Table 1:** Symptoms of spinal cord compression by tumour location.

Metastasis Location	Signs and Symptoms
Cervical spine	Headache, neckache, shoulder/arm pain Loss of upper limb sensation Weakness of neck, trunk, and upper limb muscles Paresis/paralysis involving the neck, trunk, and upper limbs
Thoracic spine	Chest and/or back pain Decreased sensation below the tumour level/Increased sensation above the tumour level Muscle weakness below the tumour level Paresis/paralysis Bladder, bowel, sexual dysfunction Positive Babinski reflex
Lumbosacral spine	Low back pain ± radiculopathy Lower limb muscle weakness Lower limb muscle paresis/paralysis Decreased or absent lower limb reflexes Drop foot Decreased or absent lower limb reflexes Bladder, bowel, sexual dysfunction Cauda equina syndrome—perineal/perianal loss of sensation (“saddle anaesthesia”), loss of rectal tone, absent bulbocavernosus, and anal wink reflexes

## Data Availability

All raw data are available to access should they be requested.
